# Design and implementation of a standard care programme of therapeutic exercise and education for breast cancer survivors

**DOI:** 10.1007/s00520-021-06470-9

**Published:** 2021-08-31

**Authors:** Cristina Roldán-Jiménez, Bella Pajares, Sofía Ruiz-Medina, Manuel Trinidad-Fernández, Manuel González-Sánchez, Nuria Ribelles, José Manuel García-Almeida, María José Ríos-López, Emilio Alba, Antonio Ignacio Cuesta-Vargas

**Affiliations:** 1 Departamento de Fisioterapia, Facultad de Ciencias de La Salud, Universidad de Málaga, Andalucia Tech, Málaga, Spain; 2 Instituto de Investigación Biomédica de Málaga (IBIMA), Málaga, Spain; 3 Hospital Universitario Virgen de La Victoria, Málaga, Spain; 4 School of Clinical Science, Faculty of Health Science, Queensland University Technology, Brisbane, Australia

**Keywords:** Breast cancer, Breast cancer survivors, Community, Exercise therapy, Nutrition therapy

## Abstract

**Background:**

Breast cancer survivors (BCS) face several symptoms and are at higher risk of weight gain following diagnosis. Current literature shows that both exercise and diet play a key role in recovery of BCS. However, there is a gap between current guidelines and the real-world context. The aim of this article is to describe the process behind a free, not-for-profit community-based therapeutic exercise and education programme (TEEP) for BCS in the clinical setting.

**Methods:**

The “Onco-Health Club” (OHC) consists of therapeutic exercise (TE) intervention aimed at ameliorating cancer-related fatigue (CRF) and improving QoL and physical function. TE is supplemented with nutritional education, providing information about the Mediterranean diet. To this end, patients are recruited from an oncologist and are referred to a physiotherapist and a nutritionist for baseline assessment. TEEP consists of a 3-month intervention, delivered twice a week in a group format with 1 h of TE and 30 min of nutritional education. BCS then have a final assessment and are advised to continue with a healthy lifestyle. Data about referral, compliance and assessment were collected.

**Results:**

From May 2017 to February of 2020, a total of 158 patients were recruited from 8 cohorts and 142 initially started the OHC. From 119 that joined the program, 96 patients were considered to have finished it with good adherence (assistance > 80%). BCS significantly improved their QoL, as well as upper and lower limb’s function, and increased their level of physical activity. CRF tended to decrease (*p* = 0.005).

**Conclusions:**

This study obtained data on recruitment, compliance, and possible limitations of these kinds of programmes in a real-world context. Further research is needed in order to optimize patient engagement and compliance, as well as to determine the transferability of these programmes in the clinical setting.

**Trial registration:**

NCT03879096, Registered 18th March 2019. Retrospectively registered.

**Supplementary Information:**

The online version contains supplementary material available at 10.1007/s00520-021-06470-9.

## Background

Breast cancer (BC) is the most commonly diagnosed type of cancer in women, expecting to account for 30% of all new cancer diagnosis in different allocations. The death rate in patients with BC has decreased by 38% over recent decades [1], with current 5-year survival rates of 90%. This translates into an increasing number of breast cancer survivors (BCS) worldwide [2].

During the survivorship period, BCS face several symptoms and side effects, such as cancer-related fatigue (CRF), anxiety, depression, altered sleep quality, or cancer-related lymphedema [3]. BCS have also impaired quality of life (QoL) and physical function. Physical exercise for therapeutic proposes, namely therapeutic exercise (TE), has proven to ameliorate this impairment [3]. Moreover, physically active BCS have a 30–50% reduced risk of disease recurrence and mortality [4]. This is why current guidelines point to TE as a crucial intervention to be included as part of standard cancer care [5–11].

In addition to exercise, diet plays a leading role in BC survivorship: the promotion of a healthy diet might reduce the risk of recurrence [12] and symptoms [13]. The American Cancer Society (ACS) guidelines for cancer survivors recommend regularly consuming vegetables, fruits, and whole grains; achieving and maintaining a healthy weight; and engaging in regular physical activity [14]. Specifically, weight gain is associated with an increase in all-cause mortality in BCS [15].

Despite current recommendations, BCS do not achieve the minimal requirements of physical activity [16] and have barriers to exercise [17]. There is a gap between literature and clinical practice, where there is a lack of implementation of standard care programmes. Research in a real-life context is needed to determine the transferability of these programmes. The aims of this article were therefore (a) to describe the process behind a free, not-for-profit community-based therapeutic exercise and education programme (TEEP) for BCS in the clinical setting in terms of design, participants referral and eligibility, beginning and baseline assessment, intervention and funding, and sustainability; and (b) to determine the recruitment, the compliance and the improvement in outcomes after its completion.

## Methods

### Programme design and description

The *School of Healthy Habits for Women Operated for Breast Cancer*, known colloquially as the *Onco-Health Club* (OHC), started out in May 2017. This community programme is delivered at University Clinical Hospital Virgen de la Victoria (Málaga, Spain). The OHC started out as part of a research network between the Translation Research in Cancer B-01 and Clinimetric F-14 research groups at Málaga Biomedical Research Institute (IBIMA), accredited for healthcare research in Spain by Carlos III Institute of Health (www.ibima.eu/en).

The main goal of OHC is to transfer the current guidelines on exercise and diet in the oncology field to the clinical setting. BCS can therefore benefit from the effect of TE interventions, such as ameliorating CRF and improving QoL and physical function. The inclusion of nutritional education aims to provide information about the Mediterranean diet. The goal of this combination of interventions is to empower patients and provide practical tools to maintain a healthy lifestyle after this 12-week programme.

### Participant referral and eligibility

Women were initially referred to the OHC by Medical Oncologists from the Medical Oncology Unit at the hospital. From the OHC, participants were then referred to a range of sources, such as the Málaga Breast Cancer Association (ASAMMA), oncologists from other hospitals, or talks given for cancer patients by oncologists (BCP, EA) and physiotherapists (ACV, CRJ), organized by associations or the University of Málaga.

The term cancer survivor applies to any individual from the time of diagnosis, during and immediately after treatment, who is still living [18]. However, to be eligible for the OHC, women must have been surgically treated for their primary tumour with no evidence of recurrence, presence of tumour, or metastatic disease at the time of recruitment. Patients were excluded if they had suffered any cardiovascular event defined as stable or unstable angina, acute pulmonary oedema, cardiac rhythm disorders, or syncope of unrelated aetiology in the year prior to inclusion. Patients were also excluded if they were already taking regular exercise.

### Beginning the programme and baseline assessment

After confirmation of eligibility, participants in this study signed an informed consent form. The oncologists collected clinical data on family history, comorbidities, cardiovascular risk factors, surgical interventions, and musculoskeletal system pathology. Participants then attended physical medicine and rehabilitation outpatient sessions at the hospital for assessment. During these visits, a physiotherapist (CRJ) carried out a clinical interview and a physical assessment. Patients reported their clinical history in the clinical interview, thus ensuring personalized intervention based on the clinical information facilitated by the oncologist and the details of the interview [7, 19]. The physical assessment was used to check musculoskeletal signs and symptoms, range of motion limitations, and motor control. Patients underwent a fitness test, which consisted of a submaximal oncology ergometry following a protocol for BCS [20]. More details are given in additional file 2.

Finally, a functional assessment was provided by 30-STS [21], hand-grip strength [22], and the following questionnaires: Piper Fatigue Scale-Revised (PFS-R) [23], the Upper Limb Functional Index (ULFI) [24], the Lower Limb Functional Index (LLFI) [25], the International Physical Activity Questionnaire-Short Form (IPAQ-SF) [26], the *European Organisation for Research and Treatment of Cancer Quality of Life Questionnaire Core 30* (EORTC QLQ-C30) [27], and the *European Organisation for Research and Treatment of Cancer Breast Cancer-Specific Quality of Life questionnaire* (EORTC QLQ-BR23) [28].

In the visit, a nutritionist (JMRL) assessed nutritional habits and the food consumed during the previous 24 h in order to specifically target the nutritional education. Adherence to Mediterranean diet was measured by the Mediterranean Adherence Screener Score [29]. Body composition was also analyzed.

More details about baseline assessment are provided in additional file 1.

### Therapeutic exercise intervention

Participants underwent an individually tailored TE intervention based on their clinical history and baseline physical and fitness assessment. More details are given in additional file 2.

### Educational intervention

Nutritional education was carried out prior to exercise. Sessions were individualized based on a clinical interview, which included body composition analysis and eating habits. Each session consisted of 30 min of nutrition information about the Mediterranean diet, macronutrients, food properties, meal distribution, calorie intake, and myths about food and cancer.

### Funding and sustainability

OHC is partially funded by Contract No. PS16060 in IBIMA between Novartis-IBIMA. This funding consists of a payment for CRJ as the physiotherapist and MJRL as the nutritionist. The Málaga Breast Cancer Association (ASAMMA) also made a donation that helped ensure continuity. The Chair of Physiotherapy at University of Málaga provided material for assessment and inventory material. The University Clinical Hospital Virgen de la Victoria provided the rehabilitation room equipped with treadmills, bicycles, dumbbells, weights, and mats.

Sustainability of the programme also depends on cooperation and collaboration with oncologists from local hospitals who voluntarily screen eligibility criteria and facilitate contact between patients and clinicians from the TEEP, as well as provide oncology data to the physiotherapist. Referral by the oncologist plays an important role in recruitment.

### Statistical analysis.

To measure the effect of the TEEP, differences between baseline and final assessment were calculated by ANOVA (*F*, *p*). All statistical analyses were conducted using SPSS 22.0 for Windows.

## Results

### Recruitment

**Fig. 1:**
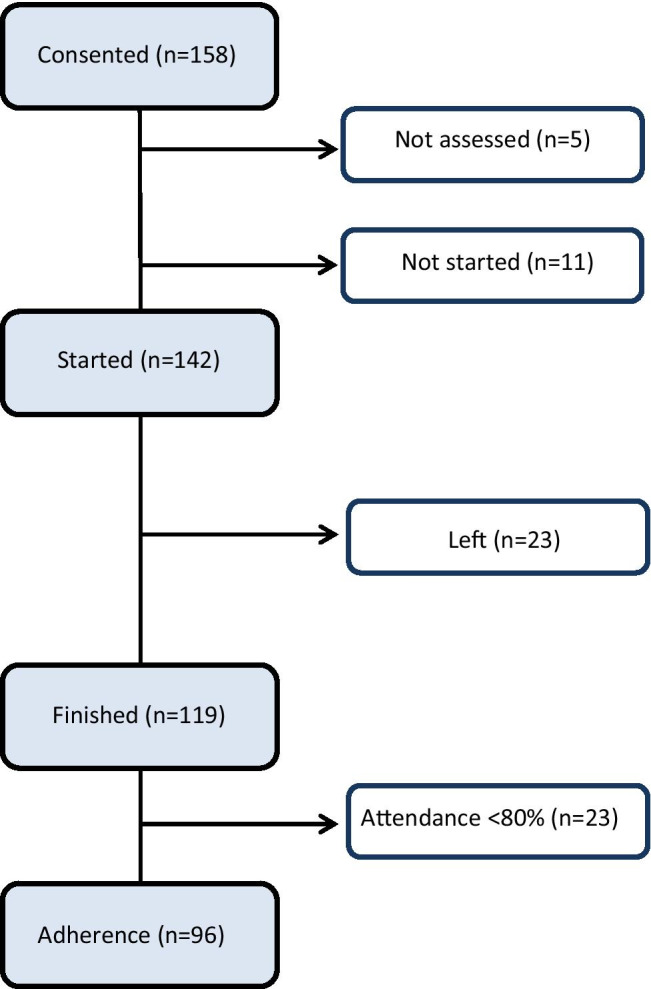
Recruitment process and compliance in the Onco-Health Club programme

The recruitment process and compliance are summarized in Fig. 1.

During the programme, participants attended the hospital twice a week, every Tuesday and Thursday evening. Each cohort was organized in two groups of 10–12 patients. The intervention took 3 months, with a slight break to take measurements between each cohort.

### Compliance

From May 2017 to February of 2020, a total of 158 patients were recruited from 8 cohorts. Once the informed consent was signed, 5 of them did not attend the visit for assessment due to incompatibility with their work, personal matters, or unknown reasons. After baseline assessment, 11 of them did not start the TEEP because of unknown reasons (it was impossible get in touch with them again). Of 142 patients who joined the OHC, 23 withdrew due to the reasons detailed in Table 1.

**Table 1:** Reasons reported by patients to withdraw from the Onco-Health Club

Incompatibility with work (3)	Change in work shift, overload (2)
Incompatibility with family life (3)	Care of children, care of parent (2)
Health problems (12)	Mastitis, breast surgery with expander, conjunctivitis, flu (3), radiation burn, fibula fracture, haematuria, eye cataract surgery, pneumonia, visual impairments due to medication
Transport barriers (2)	Family member cannot bring them by car, impossible to take public transport after knee injury
Others (3)	Contraindication by radiologist, lack of interest, unknown

### Number of patients reported between brackets when higher than 1

Of the remaining 119 patients, 23 had attendance of less than 17 days, representing an attendance rate of less than 80% of days. Lack of attendance was mostly due to incompatibility with work, family care issues, and visits to a healthcare professional (oncology revision or dentist). Some of them reported specific health problems such as breast implant surgery and fear of recurrence after breast biopsy. As a result, 96 were considered to have finished the programme with good adherence (Fig. 1), representing approximately 67% of the 142 who started the TEEP. Of those with good adherence, 64 women (66.6%) had full attendance, 19 women (19.7%) had absence of attendance of 4 days or less, and 13 women (13.5%) had absence of attendance of over 4 days.

### Improvements in outcomes

Differences between baseline and final assessment were calculated in those patients with good adherence (rate of more than 80% of days), as a low number of sessions may have negatively influenced results. Furthermore, only 6 out of 23 patients with low adherence (rate of less than 80% of days) completed the final assessment. The description of the whole sample and the improvements in physical and nutritional outcomes are presented in additional file 3.

### Final assessment

The women undergo a final assessment after the TEET intervention, so possible improvements in outcomes could be measured. In this final assessment, the physiotherapist (CRJ) and the nutritionist (MJRL) advise patients to continue with their active lifestyle in other settings. An oncologist (BP) closes the OHC programme with an educational talk recalling everything learned, and contact is made with other patients’ associations. In this talk, patients from prior cohorts are invited and given an individual report with the changes in the assessment results during the TEEP. There is therefore a short period of time between each cohort for recruitment and assessment purposes.

**Fig. 2:**
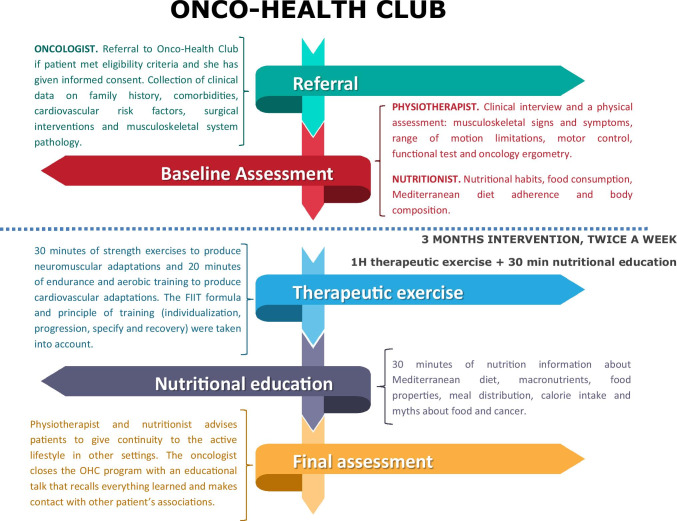
Implementation Scheme of Onco-Health Club programme

Implementation of the Onco-Health Club is summarized in Fig. 2.

## Discussion and conclusion

The TEEP from the OHC provides BCS with a standard care intervention through a tailored, supervised healthy lifestyle environment led by health professionals. This ongoing programme represents a transfer of research findings to the clinical field, breaking down the gap between evidence of TEEP benefits and late implementation in clinical practice [30, 31]. One of the strengths of TEEP is that the exercise intervention envisages the FITT training principles, in addition to individualization, progression, specificity, and recovery (see additional file 1). In the oncology setting, exercise prescription is limited by relatively generic exercise guidelines, and there is special interest in exercise individualization and guidance on exercise dose in order to fulfil the requirements for therapeutic effectiveness [32, 33]. Another strength is that, besides oncology history and clinical interview, the baseline assessment includes measures widely employed in the research field, such as hand-grip strength [34, 35], 30-STS [20, 36], and validated patient-reported outcomes (additional file 2). This allowed changes to be measured in several outcomes. As a result, women who joined the OHC decreased their level of fatigue, improved their physical function, increased their level of physical activity, and improved their adherence to the Mediterranean diet (additional file 3). Given that empowerment and staff support are important for cancer patients to gain control over their health and return to their normal functioning [37], another strength is that the TEEP ensured learning and behaviour change, and it was closed with an educational talk for positive long-term behaviour.

The OHC takes place in Málaga (Spain). A total of 158 BCS were offered this programme between May 2017 and February 2020. The prevalence of BC in the city of Málaga ranged between 275 and 283 per year [38]. Given the high survival rates in BC, a great number of diagnosed women will face survivorship. Besides high prevalence and survival rates, the OHC was the first and only free and not-for-profit community-based TEEP developed for cancer patients in the city. This programme therefore does not meet the needs of all patients facing BC survivorship, and there is a high need for the implementation of this type of programme or service in the Public Health System. For easier replication in other settings, the baseline assessment and exercise prescription are further detailed in additional files 1 and 2.

In the research field, individualized TEEP programmes such as the OHC are recommended as part of standard care for cancer patients in order to improve outcomes and reduce cost burden [39]. It is known that the vast majority of cancer patients and survivors do not achieve the established guidelines for physical activity. This has led to the development of strategies to integrate exercise services in cancer care and to encourage oncologists to refer to rehabilitation programmes [40]. However, in the real-world, oncologists have no opportunity to refer patients to this service. Some institutions, such as the Canadian Cancer Society, are setting up grants to develop programmes in real-world settings in order to improve health outcomes for cancer survivors [41]. In Australia, some private centres have partnered with the University Research Institute to create a co-located exercise clinic to provide exercise for patients undergoing cancer treatment [42]. Despite these examples of attempts of implementation, the lack of accessible TEEP in cancer patients is still a barrier in the real-world setting [43].

### Recruitment

One strength of the present programme was the participation of an oncologist, who was the main health professional that recruited patients interested in the TEEP. Although lack of knowledge and skill about exercise prescription is considered a barrier to exercise among BCS [44], current guidelines state that oncologist assessment, advice, and referral to TEEP programmes are vital for patient engagement. Referral to rehabilitation healthcare professionals for further evaluation allows us to break down this barrier [40]. In the OHC programme, communication between the oncologist and the physiotherapist allowed this multidisciplinary approach, with two-step screenings for safety. Despite 158 women signing the informed consent, 5 of them did not attend the baseline assessment and 11 of them dropped out after assessment. As TEEP was offered for free, this may have been due to patients not being responsible enough in terms of attendance and commitment to TEEP. If this programme were to become part of the Public Health System in the future, it would be advisable to have an assistant to carry out a screening with a motivational interview in order to ensure the patient is genuinely interested, thus reducing the likelihood of lack of attendance.

### Compliance

Another strength of this study is that days of attendance are reported in terms of compliance [45]. It should be noted that only 67% of women who joined the TEEP finished it with good adherence. Treatment-related side effects, lack of time, and CRF are the main barriers to exercise in this population. Interventions such as the presented TEEP would therefore benefit from motivational interviews for time management, behavioural change techniques, and support and education about the effects of exercise on CRF and treatment-related side effects [17]. Lack of attendance due to health problems also suggests these community-based programmes should be continued over time, as the patient can be expected to be active with physical limitations and some kind of supervision.

It should be highlighted that the women who joined this programme were interested in exercise and nutrition habits. However, the proportion of women who would have refused to participate if this service were offered is unknown. In other cancer populations, such as patients with advanced disease, only 77 of 524 agreed to join a 6-week home-based workout programme [46]. Previous research has also studied the influence of outcomes such as sense of coherence, QoL, and demographic and surgical-related outcomes with these programmes [47, 48]. To successfully implement TEEP programmes in the real-world setting, further research is needed on strategies to modify patient perception of exercise, how to engage them in these programmes, and outcomes related to compliance.

Given the COVID-19 pandemic, future programmes should be oriented towards mixed or bi-modal interventions which include both face-to-face and online supervision depending on patient’s preferences [49] and demographics [50]. In addition, implementing technology-supported interventions [51] may allow to offer this service to a greater number of patients in those cases in which supervision is less required [52]. In addition, the opportunity to deliver the OHC online may facilitate reported barriers such as incompatibility with work (Table 1), which has also shown to be a predictor for low attendance [53].

## Conclusions

The Onco-Health programme provided a TEEP intervention for BCS based on clinical oncology guidelines. This allowed data to be obtained about recruitment, compliance, and possible limitations of these kinds of programmes in a real-world context. BCS who completed the programme with good adherence decreased their level of fatigue, improved their physical function, increased their level of physical activity and improved their adherence to the Mediterranean diet. Further research is needed in order to optimize patient engagement and compliance, as well as to determine the transferability of these programmes in the clinical setting.

## Supplementary Information

Below is the link to the electronic supplementary material.

Supplementary file1 (DOCX 39 KB)

Supplementary file2 (DOCX 30 KB)

Supplementary file3 (DOCX 24 KB)

Supplementary file4 (DOC 221 KB)

## Data Availability

The datasets analyzed during the current study are available from the corresponding author on reasonable request.
