# Supplementary material for: Design and implementation of a standard care programme of therapeutic exercise and education for breast cancer survivors
Source: Support Care Cancer. 2021 Aug 31;30(2):1243–51. doi: 10.1007/s00520-021-06470-9 (PMC8405716; doi:10.1007/s00520-021-06470-9)
Supplement: Supplementary file 1 — Supplementary file1 (DOCX 39 KB) [file 520_2021_6470_MOESM1_ESM.docx]

**Additional File 1: Baseline assessment by physiotherapist and nutritionist.**

The physiotherapist measured the following outcomes:

- *Handgrip Strength*. Jamar Hydraulic Hand Dynamometer Model SH5001 [Lafayette Instrument, Lafayette, USA] was used to record strength. Handgrip dynamometry is the main measure of grip strength [1]. All participants sat on a chair without armrests with their feet on the floor and a straight back, holding the dynamometer with the dominant arm. The elbow was flexed at 90° and the wrist was in neutral position (0º). The participant performed three isometric maximal voluntary contractions for 5 seconds. Strength was expressed in kg [2].
- *Cancer-Related Fatigue* (CRF). The Spanish version of the PFS-R was used. This contains 22 items with scores ranging from 0 to 10 and includes four aspects of subjective fatigue: behavioural/severity (6 items), affective meaning (5 items), sensory (5 items) and cognitive/mood (6 items). This scale has high reliability (Cronbach’s α=0.96) in this population [3]. To obtain scores for each subjective domain of fatigue, the score from each item (0-10) was multiplied by its load factor. The sum of all domains provided a total score that ranged from 0 to 118.41, in accordance with psychometric properties [4]. Another total score was calculated as the overall sum (0-220). This total score was also transferred to a 0-10 point scale (0 = none, 1–3 = mild, 4–6 = moderate, 7–10 = severe) [5], with higher scores indicating greater fatigue [3]. A change of one unit in the fatigue score (e.g. 5–4) represents a clinically significant change in fatigue [6]. These scores are comparable to other research and its clinical application.
- *Upper limb functionality*: the Spanish version of Upper Limb Functional Index (ULFI) questionnaire was filled in by each participant. ULFI is an upper extremity outcome measure that consists of a 25-item scale transferable to a 100-point scale. It also has strong psychometric properties for reliability and validity [7]**.** Values are expressed as a functionality percentage (%).
- *Lower limb functionality*: the Spanish version of the Lower Limb Functional Index (LLFI) questionnaire was filled in by each participant. LLFI is an upper extremity outcome measure that consists of a 25-item scale transferable to a 100-point scale. It also has strong high internal consistency (α = 0.91) and high reliability (ICC = 0.96) [8]. Values are expressed as a functionality percentage (%).
- *Physical activity:* The International Physical Activity Questionnaire-Short Form (IPAQ-SF) questionnaire, which asks about three specific types of activity (walking, moderate-intensity activities, and vigorous-intensity activities) over the last 7 days. Values are expressed in METS. IPAQ-SF presents acceptable measurement properties, comparable to other self-reported physical activity measurements [9].
- Quality of life (QoL) was assessed by two questionnaires:
- The European Organization for Research and Treatment of Cancer Quality of Life Questionnaire Core 30 *(EORTC QLQ-C30)*version 3*.*0 [10] is a cancer health-related QoL questionnaire. EORTC QLQ-C30 comprises 30 items and contains five functional scales, three symptom scales, a global health status/QoL scale, and six single items. Raw scores can be linearly converted to a 0–100 scale with higher scores reflecting higher levels of function and higher levels of symptom show bigger problems. [11]. The Spanish version has shown to be a valid reliable questionnaire [12].
- The European Organization for Research and Treatment of Cancer Breast Cancer-Specific Quality of Life questionnaire (EORTC QLQ-BR23)*.* This is a breast cancer module of EORTC QLQ-C30 which contains 23 items that assess disease symptom, side effects of treatment, body image, sexual functioning, and future perspective. All items are rated on a 4-point scale (from 1- not at all, to very much). The scoring procedure is the same as EORTC QLQ-C30 [11]. Higher scores represent better functioning, and higher scores of symptoms show bigger issues. Cronbach’s α ranged between 0.46-0.94, showing a high reliability [10].

The nutritionist measured the following outcomes:

- *Body Composition*. We measured BMI, basal metabolism, fat/lean mass and water balance. Bioelectrical impedance was analysed using Tanita TBF-300A (TANITA CORPORATION, Tokyo, Japan). This involves impedance measurement with a Tetrapolar Bioelectrical Impedance Analysis system between both feet, with a frequency of 50kHz, a measurement current of 500 μA and a range of 150 ~ 900Ω. Furthermore, impedance, weight (kg), body mass index, basal metabolism in kJ, basal metabolism in Kcal, fat mass (Kg), lean mass (kg) and total amount of water in the body (kg) were all measured.
- *Mediterranean diet adherence:* We used a 14-point Mediterranean Adherence Screener score. This questionnaire is a modification of a previous 9-item questionnaire, in which the score was inversely associated with mortality from cancer and other chronic diseases [13]. The 9-item questionnaire incorporates the inherent characteristics of Mediterranean dietary patterns: non-refined cereals, fruit, vegetables, potatoes, pulses, olive oil, fish, red meat, poultry, full-fat dairy products and alcohol. 5 additional items assess adherence to traditional Mediterranean diet among the Spanish population. Items 1 and 13 use short questions to inquire about food habits, while items 7, 12 and 14 are questions about food consumption frequency for nuts, soda drinks and a typical Mediterranean sauce made of tomato, garlic, onion, or leeks sautéed in olive oil (known as “*sofrito*”) [14]. The validity of the Mediterranean Adherence Screener Score has been studied in the Spanish population [15].

Table shows criteria for 1 point in each item. Total scores ranges from 0 (minimal adherence) to 14 (maximal adherence). Adherence can be stratified based on scores as low (≤7 points), medium (8-9 points), and high (≥10 points). Medium and high adherence levels are associated with a lower risk of developing head and neck cancer [16].

| **Questions** | **Criteria for 1 point** |
| --- | --- |
| 1. Do you use olive oil as main culinary fat? | Yes |
| 2. How much olive oil do you consume in a given day (including oil used for frying, salads, out-of-house meals, etc.)? | ≥4 tbsp |
| 3. How many vegetable servings do you consume per day? (1 serving : 200 g [consider side dishes as half a serving]) | ≥2 (≥1 portion raw or as a salad) |
| 4. How many fruit units (including natural fruit juices) do you consume per day? | ≥3 |
| 5. How many servings of red meat, hamburger, or meat products (ham, sausage, etc.) do you consume per day? (1 serving: 100–150 g) | <1 |
| 6. How many servings of butter, margarine, or cream do you consume per day? (1 serving: 12 g) | <1 |
| 7. How many sweet or carbonated beverages do you drink per day? | <1 |
| 8. How much wine do you drink per week? | ≥7 glasses |
| 9. How many servings of legumes do you consume per week? (1 serving : 150 g) | ≥3 |
| 10. How many servings of fish or shellfish do you consume per week? (1 serving 100–150 g of fish or 4–5 units or 200 g of shellfish) | ≥3 |
| 11. How many times per week do you consume commercial sweets or pastries (not homemade), such as cakes, cookies, biscuits, or custard? | <3 |
| 12. How many servings of nuts (including peanuts) do you consume per week? (1 serving 30 g) | ≥3 |
| 13. Do you preferentially consume chicken, turkey, or rabbit meat instead of veal, pork, hamburger, or sausage? | Yes |
| 14. How many times per week do you consume vegetables, pasta, rice, or other dishes seasoned with sofrito (sauce made with tomato and onion, leek, or garlic and simmered with olive oil)? | ≥2 |

**References**

1. Roberts HC, Denison HJ, Martin HJ, Patel HP, Syddall H, Cooper C, et al. A review of the measurement of grip strength in clinical and epidemiological studies: towards a standardised approach. Age Ageing. 2011;40:423–9.

2. Trinidad-Fernández M, González-Molina F, Moya-Esteban A, Roldán-Jiménez C, González-Sánchez M, Cuesta-Vargas AI. Muscle activity and architecture as a predictor of hand-grip strength. Physiol Meas. 2020;41:075008.

3. Piper BF, Dibble SL, Dodd MJ, Weiss MC, Slaughter RE, Paul SM. The revised Piper Fatigue Scale: psychometric evaluation in women with breast cancer. Oncol Nurs Forum. 1998;25:677–84.

4. Cantarero-Villanueva I, Fernández-Lao C, Díaz-Rodríguez L, Cuesta-Vargas AI, Fernández-de-las-Peñas C, Piper BF, et al. The Piper Fatigue Scale-Revised: translation and psychometric evaluation in Spanish-speaking breast cancer survivors. Qual Life Res Int J Qual Life Asp Treat Care Rehabil. 2014;23:271–6.

5. Mock V. Clinical excellence through evidence-based practice: fatigue management as a model. Oncol Nurs Forum. 2003;30:787–96.

6. Schwartz AL, Meek PM, Nail LM, Fargo J, Lundquist M, Donofrio M, et al. Measurement of fatigue. determining minimally important clinical differences. J Clin Epidemiol. 2002;55:239–44.

7. Cuesta-Vargas AI, Gabel PC. Cross-cultural adaptation, reliability and validity of the Spanish version of the upper limb functional index. Health Qual Life Outcomes. 2013;11:126.

8. Cuesta-Vargas AI, Gabel CP, Bennett P. Cross cultural adaptation and validation of a Spanish version of the Lower Limb Functional Index. Health Qual Life Outcomes. 2014;12:75.

9. Craig CL, Marshall AL, Sjöström M, Bauman AE, Booth ML, Ainsworth BE, et al. International physical activity questionnaire: 12-country reliability and validity. Med Sci Sports Exerc. 2003;35:1381–95.

10. Sprangers MA, Groenvold M, Arraras JI, Franklin J, te Velde A, Muller M, et al. The European Organization for Research and Treatment of Cancer breast cancer-specific quality-of-life questionnaire module: first results from a three-country field study. J Clin Oncol. 1996;14:2756–68.

11. Fayers PM. EORTC QLQ-C30 Scoring Manual. 2001.

12. Arraras JI, Arias F, Tejedor M, Pruja E, Marcos M, Martínez E, et al. The EORTC QLQ-C30 (version 3.0) Quality of Life questionnaire: validation study for Spain with head and neck cancer patients. Psychooncology. 2002;11:249–56.

13. Trichopoulou A, Costacou T, Bamia C, Trichopoulos D. Adherence to a Mediterranean Diet and Survival in a Greek Population. http://dx.doi.org/10.1056/NEJMoa025039. 2009. doi:10.1056/NEJMoa025039.

14. Martínez-González MA, García-Arellano A, Toledo E, Salas-Salvadó J, Buil-Cosiales P, Corella D, et al. A 14-Item Mediterranean Diet Assessment Tool and Obesity Indexes among High-Risk Subjects: The PREDIMED Trial. PLoS ONE. 2012;7. doi:10.1371/journal.pone.0043134.

15. Schröder H, Fitó M, Estruch R, Martínez-González MA, Corella D, Salas-Salvadó J, et al. A short screener is valid for assessing Mediterranean diet adherence among older Spanish men and women. J Nutr. 2011;141:1140–5.

16. Salvatore Benito A, Valero Zanuy MÁ, Alarza Cano M, Ruiz Alonso A, Alda Bravo I, Rogero Blanco E, et al. Adherence to Mediterranean diet: A comparison of patients with head and neck cancer and healthy population. Endocrinol Diabetes Nutr. 2019;66:417–24.
