# Supplementary material for: Design and implementation of a standard care programme of therapeutic exercise and education for breast cancer survivors
Source: Support Care Cancer. 2021 Aug 31;30(2):1243–51. doi: 10.1007/s00520-021-06470-9 (PMC8405716; doi:10.1007/s00520-021-06470-9)
Supplement: Supplementary file 2 — Supplementary file2 (DOCX 30 KB) [file 520_2021_6470_MOESM2_ESM.docx]

**Additional File 1: Therapeutic exercise**

Exercises to generate neuromuscular adaptations and cardiovascular adaptations were based on the protocol described by Andersen and Aagaard [1], accounting for the principles of training [2] and current recommendations in the oncology field [3].

The next FITT formula [2] was followed**:**

- Frequency: 24 sessions.
- Intensity: Moderate; more details are given along with this file.
- Time: A total of 1 hour per session (2 hours per week). 30 minutes of strength exercises, followed by 20 minutes of endurance and aerobic training.
- Type: Exercise modalities consisted of muscular strength training (30 minutes) to induce neuromuscular adaptations with endurance and aerobic training (20 min) to induce cardiovascular adaptations. Warm-up and cool-down exercises were included.

**Neuromuscular adaptations**

*Individualization*: The intervention was preceded by a physical assessment of the musculoskeletal system to assess any impaired range of motion and motor control. These were used to establish individualised adaptations, loads and targeted muscle groups.

*Progression*:

| **Week** | **Objective** | **Method** |
| --- | --- | --- |
| **1–2** | Learn proper exercise technique | Patients carried out 3 sets of 15 repetitions (reps) with a load that would guarantee proper execution [4].. In the upper limbs, elastic bands were used if they could not achieve the goal with a weight of 0.5 kg and/or if the patient presented with kinesiophobia. |
| **2–4** | Target exercise dose | Patients carried out 4 sets of 10 reps. If the patient could perform more than 12 reps with the proper execution, the weight to be lifted was increased [4]*.* |
| **12–14** | Ensure learning and behavior change | Patients were informed about changes and progression made since the beginning to make them conscious about their improvement. Personal adaptations and self-perceptions were discussed to empower patients for positive long-term exercise behaviour, |

In order to ensure proper technique and adequate progression, all exercises and changes were incorporated and supervised by a physical therapist.

*Specificity*: Muscular strength training was carried out in order to obtain adaptation in the musculoskeletal system and improve function. Exercises targeted the major muscle groups according to the literature [5]. Muscle isolation was carried out as determined by muscle weakness and patient priorities during the physical assessment.

*Recovery****:*** To optimize physiological adaptations, sessions were held on Tuesdays and Thursdays, with a minimum of 48 hours of recovery between sessions. Furthermore, patients were asked to report any symptom days following exercise to the physiotherapist in order to avoid overload.

**Cardiorespiratory adaptations**

*Individualization*: The aerobic-anaerobic transition zone was determined [4]. For this purpose, an oncology ergometry was carried out based on heart rate (HR) [6] and perceived exertion (PE). The fitness test was performed on a treadmill BH Mercury Max G6507 (BH FITNESS); heart rate was obtained with a Polar pulsometer M400 with a Thoracic band H7 HR (POLAR Spain) and perceived exertion was measured with the Borg Scale [7]. This submaximal aerobic test is carried out on a treadmill, and each stage lasts 2 minutes. The speed is gradually increased till the 85% of the maximum age-predicted heart rate (MPHR) is reached. This oncology ergometry allows estimating % of MPHR and the perceived exertion for prescription purposes in BCS [8].

*Progression*:

| **Week** | **Objective** | **Method** |
| --- | --- | --- |
| **1–2** | Adapt to the experience of fatigue during exercise. | low-intensity adaptation (under 60% HR). |
| **2–14** | maintain a constant  speed to achieve the prescribed aerobic heart rate thresholds based on an individualised test [9]. | Patients were told to maintain both the velocity and PE corresponding to a range between 60% and 80% of their maximum HR. If experiencing any symptoms while exercising, patients reduced intensity.  Every two weeks, HR and PE at a selected velocity were measured in order to increase intensity in cases of improvement. |
| **12–14** | Ensure learning and behavior change | These were the same methods as those of the neuromuscular adaptations. |

*Specificity*: Endurance with aerobic training was carried out at moderate intensity. During the assessment, 60% and 80% of HR was correlated with the treadmill velocity and PE. This allowed feedback maintenance between the physiotherapist and the patient during the program to achieve the proper exercise dose.

*Recovery****:*** The same method was used as the recovery method for neuromuscular adaptations.

**References**

1. Andersen JL, Aagaard P (2000) Myosin heavy chain IIX overshoot in human skeletal muscle. Muscle Nerve 23:1095–1104

2. Sasso JP, Eves ND, Christensen JF, et al (2015) A framework for prescription in exercise-oncology research. J Cachexia Sarcopenia Muscle 6:115–124. https://doi.org/10.1002/jcsm.12042

3. Dennett AM, Peiris CL, Shields N, et al (2016) Moderate-intensity exercise reduces fatigue and improves mobility in cancer survivors: a systematic review and meta-regression. J Physiother 62:68–82. https://doi.org/10.1016/j.jphys.2016.02.012

4. Cuesta-Vargas AI, Carabantes F, Caracuel Z, et al (2016) Effectiveness of an individualized program of muscular strength and endurance with aerobic training for improving germ cell cancer-related fatigue in men undergoing chemotherapy: EFICATEST study protocol for a randomized controlled trial. Trials 17:8. https://doi.org/10.1186/s13063-015-1143-x

5. Cormie P, Atkinson M, Bucci L, et al (2018) Clinical Oncology Society of Australia position statement on exercise in cancer care. Med J Aust 209:184–187

6. Conconi F, Grazzi G, Casoni I, et al (1996) The Conconi Test: Methodology After 12 Years of Application. Int J Sports Med 17:509–519. https://doi.org/10.1055/s-2007-972887

7. Borg GA (1982) Psychophysical bases of perceived exertion. Med Sci Sports Exerc 14:377–381

8. Cuesta-Vargas, Antonio Ignacio (2019) Energy System Assessment in Survivors of Breast Cancer. Physical Theraphy Journal Special Issue:

9. Cuesta-Vargas AI, Buchan J, Arroyo-Morales M (2014) A multimodal physiotherapy programme plus deep water running for improving cancer-related fatigue and quality of life in breast cancer survivors. Eur J Cancer Care (Engl) 23:15–21. https://doi.org/10.1111/ecc.12114
