# Supplementary material for: Design and implementation of a standard care programme of therapeutic exercise and education for breast cancer survivors
Source: Support Care Cancer. 2021 Aug 31;30(2):1243–51. doi: 10.1007/s00520-021-06470-9 (PMC8405716; doi:10.1007/s00520-021-06470-9)
Supplement: Supplementary file 3 — Supplementary file3 (DOCX 24 KB) [file 520_2021_6470_MOESM3_ESM.docx]

**Additional file 3.** Participant descriptive and clinical variables (n=96).

|  | Mean (SD) | Min-Max |
| --- | --- | --- |
| Age (years) | 51.43 (9.06) | 32.0-69.0 |
| BMI (Kg/m^2^) | 27.44 (5.35) | 20-47.7 |
| Years from diagnosis | 2.54 (5.18) | 0-13.0 |
| Surgical Intervention |  | Percentage (n) |
|  | Breast-Conserving surgery | 76.5% (73) |
|  | Mastectomy | 23.5% (23) |
| Cancer Treatment |  |  |
|  | Chemotherapy | 90.1% (87) |
|  | Radiotherapy | 97.2% (93) |
|  | Hormone Therapy | 84.5% (81) |
|  | Monoclonal Antibody | 12.3% (11) |
| Current treatment |  |  |
|  | None | 21.5% (21) |
|  | Radiotherapy | 2.9% (3) |
|  | Monoclonal antibody | 5.7% (5) |
|  | Hormone therapy | 64.3% (61) |

**Table 2** Outcomes from physical assessment pre- and post-intervention in patients with assistance >80% (n=96).

| **n=96** | Pre | Post | ANOVA (F,p) |
| --- | --- | --- | --- |
| CRF (0-10) | 5.22 (2.75) | 4 (6.57) | 8.008 (0.005) |
| ULFI (%) | 67.58 (23.16) | 78.49 (17.69) | 9.523 (0.002)* |
| LLFI (%) | 68.60 (30.66) | 84 (23.77) | 10.252 (0.002)* |
| IPAQ-SF (METS) | 4255.04 (7597.64) | 4948.38 (5193.66) | 7.834 (0.011)* |
| QLQ-C30 | 58.65 (13.49) | 56.05 (17.01) | 1.093 (0.298) |
| EORTC QLQ-BR23 | 41.86 (12.91) | 36.07 (11.32) | 35.147 (0.000)* |
| 30-STS (n) | 18.90 (5.35) | 23.39 (5.84) | 14.419 (<0.001)* |
| Handgrip (Kg) | 21.03 (5.37) | 22.73 (5.58) | 2.101 (0.149) |

^*p<0.05^ ^PFS-R Piper Fatigue Scale-Revised^*^, ULFI^* ^Upper Limb Functional Index,^ *^LLFI^* ^Lower Limb Functional Index,^ *^IPAQ-SF^* ^The International Physical Activity Questionnaire-Short Form questionnaire,^ *^30-STS^* ^30 seconds Sit-To-Stand Test, EORTC QLQ-C30 The European Organization for Research and Treatment of Cancer Quality of Life Questionnaire Core 30, EORTC QLQ-BR23 The European Organization for Research and Treatment of Cancer Breast Cancer^*^-^*^Specific Quality of Life questionnaire^

**Table 3** Outcomes from nutritional assessment adherence pre and post intervention in patients with assistance >80%.

|  | Pre | Post | ANOVA: F (p) |
| --- | --- | --- | --- |
| BMI (Kg/m^2^) | 27.42 (5.23) | 27.47 (5.657) | 0.004 (0.829) |
| Weight (Kg) | 71.58 (14.25) | 71.08 (13.48) | 0.049 (0.826) |
| Basal Metabolism (Kj) | 5904.75 (504.46) | 5941.71(472) | 0.210 (0.648) |
| Basal Metabolism (Kcal) | 1396 (180.66) | 1420.10 (112.96) | 0.887 (0.348) |
| Fat Mass (%) | 34.40 (8.66) | 37.29 (5.69) | 2.626 (0.109) |
| Lean Mass (Kg) | 45.62 (5.31) | 45.36 (4.99) | 0.092 (0.762) |
| Water Balance (Kg) | 33.63 (3.88) | 33.47 (3.65) | 0.062(0.804) |
| Diet adherence (0-14) | 9.36 (1.85) | 11.64 (2.90) | 8.124 (0.005) |
